# A platform for automated and label-free monitoring of morphological features and kinetics of spheroid fusion

**DOI:** 10.3389/fbioe.2022.946992

**Published:** 2022-08-26

**Authors:** Thomas Deckers, Gabriella Nilsson Hall, Ioannis Papantoniou, Jean-Marie Aerts, Veerle Bloemen

**Affiliations:** ^1^ Measure, Model and Manage Bioresponses (M3-BIORES), Department of Biosystems, KU Leuven, Leuven, Belgium; ^2^ Surface and Interface Engineered Materials (SIEM), Group T Leuven Campus, KU Leuven, Leuven, Belgium; ^3^ Prometheus, Division of Skeletal Tissue Engineering Leuven, KU Leuven, Leuven, Belgium; ^4^ Skeletal Biology and Engineering Research Center, KU Leuven, Leuven, Belgium; ^5^ Institute of Chemical Engineering Sciences, Foundation for Research and Technology—Hellas (FORTH), Patras, Greece

**Keywords:** biofabrication, morphological features, automated monitoring, image analysis, spheroid fusion kinetics, machine learning, bright-field microscopy

## Abstract

Spheroids are widely applied as building blocks for biofabrication of living tissues, where they exhibit spontaneous fusion toward an integrated structure upon contact. Tissue fusion is a fundamental biological process, but due to a lack of automated monitoring systems, the in-depth characterization of this process is still limited. Therefore, a quantitative high-throughput platform was developed to semi-automatically select doublet candidates and automatically monitor their fusion kinetics. Spheroids with varying degrees of chondrogenic maturation (days 1, 7, 14, and 21) were produced from two different cell pools, and their fusion kinetics were analyzed via the following steps: (1) by applying a novel spheroid seeding approach, the background noise was decreased due to the removal of cell debris while a sufficient number of doublets were still generated. (2) The doublet candidates were semi-automatically selected, thereby reducing the time and effort spent on manual selection. This was achieved by automatic detection of the microwells and building a random forest classifier, obtaining average accuracies, sensitivities, and precisions ranging from 95.0% to 97.4%, from 51.5% to 92.0%, and from 66.7% to 83.9%, respectively. (3) A software tool was developed to automatically extract morphological features such as the doublet area, roundness, contact length, and intersphere angle. For all data sets, the segmentation procedure obtained average sensitivities and precisions ranging from 96.8% to 98.1% and from 97.7% to 98.8%, respectively. Moreover, the average relative errors for the doublet area and contact length ranged from 1.23% to 2.26% and from 2.30% to 4.66%, respectively, while the average absolute errors for the doublet roundness and intersphere angle ranged from 0.0083 to 0.0135 and from 10.70 to 13.44°, respectively. (4) The data of both cell pools were analyzed, and an exponential model was used to extract kinetic parameters from the time-series data of the doublet roundness. For both cell pools, the technology was able to characterize the fusion rate and quality in an automated manner and allowed us to demonstrate that an increased chondrogenic maturity was linked with a decreased fusion rate. The platform is also applicable to other spheroid types, enabling an increased understanding of tissue fusion. Finally, our approach to study spheroid fusion over time will aid in the design of controlled fabrication of “assembloids” and bottom-up biofabrication of living tissues using spheroids.

## Introduction

Bottom-up tissue engineering strategies, where small building blocks are self-assembled into larger structures, have recently emerged as promising approaches for the fabrication of functional tissue implants (Ouyang et al., 2020; Burdis and Kelly 2021). They exploit the inherent fusion capacity of cellular building blocks for engineering mesoscale (mm) to macroscale (cm) tissue constructs (Laschke and Menger 2017; Mironov and Visconti, 2009). These building blocks, which can range from cell sheets (Mcdermott et al., 2019) to cell-laden modules to spheroids, mature *in vitro* into microtissues and organoids which resemble the structure and function of native tissues (Hall et al., 2020; Skylar-Scott et al., 2019). For example, in skeletal tissue engineering (TE), cartilaginous microtissues are brought into contact to obtain a large cartilage-like template which is implanted at the defect site. This cartilage intermediate is then remodeled into bone through endochondral ossification, a process related to long bone fracture healing (Hall et al., 2020; Hall et al., 2021). Other examples of engineered tissues using spheroids include the liver (Yap et al., 2013), kidneys (Yang et al., 2021), and heart (Polonchuk et al., 2021). However, characterization of spheroid production and fusion still faces challenges. First, a large number of uniformly sized spheroids have to be generated. Next, these spheroids must be fused into a stable tissue construct for *in vivo* implantation. Automated imaging technologies can provide the means to acquire the information needed to address these challenges and will eventually become indispensable for quality control in spheroid production and fusion processes.

Several tools have been developed to extract fusion-related parameters. Hajdu et al., 2010 and Kim et al., 2018 manually seeded doublets one by one, and phase-contrast images were acquired to manually extract the doublet length and contact length/intersphere angles over time. In another study (Susienka et al., 2016), a fluorescence-based platform was proposed, where doublet candidates were manually selected. To study the fusion behavior, the contact length and intersphere angles were manually extracted, while the doublet roundness, length, and width were automatically computed. In summary, most fusion setups require the manual transfer of single spheroids to generate doublets or, alternatively, the manual selection of doublet candidates. Moreover, morphological feature extraction often relies on manual annotation (Hajdu et al., 2010; Omelyanenko et al., 2020; Kim et al., 2018; Kosheleva et al., 2020) or automated extraction of a limited number of features (Susienka et al., 2016; Song et al., 2019). This becomes labor-intensive and time-consuming when conducting high-throughput screenings. Other methods, developed for the characterization of single spheroids (Piccinini 2015; Grexa et al., 2021; Vinci et al., 2012), often exhibit more automation but lack important features such as the contact length, both spheroid widths, the intersphere angles, and doublet rotation, which have proven to be relevant to understand the complex mechanisms behind tissue fusion (Susienka et al., 2016). Moreover, these methods are often validated on immature spheroids.

Tissue fusion is a fundamental process in embryonic development and required for the fabrication of viable and stable tissue implants with a certain size and shape. In order to characterize and understand the events that regulate tissue fusion, high-throughput screenings that quantify morphometric details reflective of the tissue fusion process have to be performed. Several studies have investigated the influence of different factors such as ROCK inhibitor and blebbistatin (Susienka et al., 2016), spheroid size (Olsen et al., 2015), cell type (and ratio) (Susienka et al., 2016; Kim et al., 2018; Song et al., 2019), and tissue maturation (Hajdu et al., 2010; Rago et al., 2009; Omelyanenko et al., 2020; Hall et al., 2021) on fusion behavior. Moreover, *in silico* models have been developed to gain further insight into the kinetics of spheroid fusion (Kosztin et al., 2012; Ongenae et al., 2021; McCune et al., 2014). These models can benefit from dedicated *in vitro* screening studies not only to improve them but also as a basis for model validation. Although the aforementioned studies have provided some key insights into the mechanisms involved in spheroid fusion, still, relatively little is known about this process across different tissues and conditions. Moreover, tissue fusion assays can also provide information on the viability and maturity of the spheroids (Hajdu et al., 2010), thereby potentially eliminating the need for expensive, labor-intensive, and invasive assays such as live–dead stainings, histology, and gene expression. Apart from biofabrication, the concept of spheroid fusion has also been used in cancer biology to study and quantify tumor invasiveness (Kramer et al., 2013).

**FIGURE 1:**
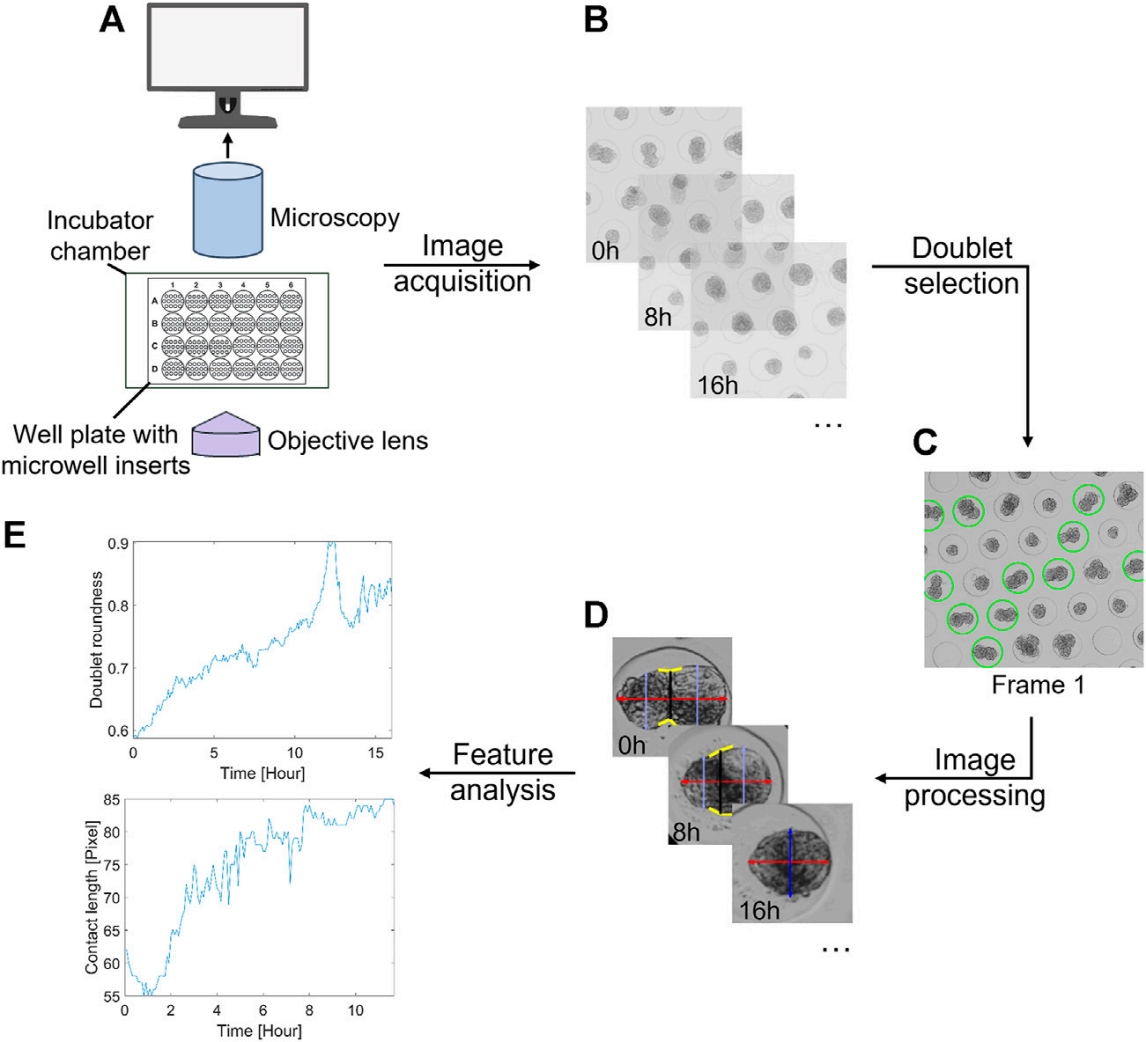
General overview of the proposed methodology. **(A)** Imaging setup, consisting of a bright-field microscope with an automated stage, Top Stage incubator, cellSens imaging software, and a 24-well plate with agarose inserts. **(B)** Acquisition of time-lapse data sets. **(C)** Manual or semi-automatic doublet selection based on the first frame. **(D)** Extraction of several morphological features over time through image analysis. **(E)** Time series analysis. As an example, the doublet roundness and contact length are displayed.

Through the deployment of automated monitoring tools, large-scale screening studies can be performed with minimal workloads. To achieve our goal, the following objectives were defined: (i) to develop a method to seed and capture doublets in a high-throughput manner, with up to 48,000 microwells per 24-well plate; (ii) to enable semi-automated selection of doublet candidates to reduce the time spent on manual labeling; and (iii) to perform automated segmentation and feature extraction over time to characterize the fusion kinetics of spheroids. In Figure 1, an overview of the proposed methodology is shown, mainly focusing on semi-automated doublet selection and fully automated extraction of morphological features and associated kinetics during spheroid fusion. To illustrate the applicability of the software tool, a biological case study was performed, investigating the influence of tissue maturity on the fusion behavior of cartilaginous microtissues for two different cell pools.

## Materials and methods

### Cell expansion

Human periosteum-derived mesenchymal stem cells (hPDCs) were isolated from periosteal biopsies of nine donors to create two different cell pools (Y and V pool, both 10–17 years age range, five females and two males/females), as previously described by Roberts *et al.* (Roberts et al., 2015). The procedures were approved by the Ethical Committee for Human Medical Research, and patient informed consent forms were obtained. The isolated cells were cultured in T175 tissue culture flasks (Greiner Bio-One) at a seeding density of 5,700 cells/cm^2^ and sub-cultured at ± 80% confluency. A standard culture medium consisting of high-glucose GlutaMAX^TM^ Dulbecco’s modified Eagle’s medium (DMEM; Invitrogen, BE) supplemented with 10% irradiated fetal bovine serum (FBS; HyClone) and 1% antibiotic–antimycotic (100 units/ml penicillin, 100 mg/ml streptomycin, and 0.25 mg/ml amphotericin B; Invitrogen, BE) was used for expansion. At passage 8, the cells were harvested and stored at −196°C in liquid nitrogen. Prior to the fusion experiments, the cells were thawed, expanded for one passage, and harvested. At all passages, cells were harvested by trypsinisation for 10 min using TrypLE^TM^ Express (Life Technologies, United Kingdom). During cell expansion, the conditions of the incubator were actively controlled at 37°C, 90% relative humidity, and 5% CO_2_.

In this study, all experiments and methods involving these cells were performed in accordance with the relevant guidelines and regulations.

### Production of microtissues

Micro-patterned agarose inserts, as described by (Leijten et al., 2016), were fabricated using 3% (w/v) agarose (Invitrogen, Belgium). Next, the inserts were UV-sterilized for 30 min and stored at 4°C in a low-glucose medium (LG-DMEM, Gibco) supplemented with 1% antibiotic–antimycotic (100 units/ml penicillin, 100 mg/ml streptomycin, and 0.25 mg/ml amphotericin B; Invitrogen). Microspheroids, composed of ≈100 cells, were obtained by seeding 200,000 cells per well and differentiated into microtissues in a serum-free chemically defined chondrogenic medium (CM) containing LG-DMEM (Gibco) supplemented with 1% antibiotic–antimycotic (100 units mL^−1^ penicillin, 100 mg ml^−1^ streptomycin, and 0.25 mg ml^−1^ amphotericin B), 100 × 10^–9^ m dexamethasone, 1 × 10^–3^ m ascorbate-2 phosphate, 40 μg ml^−1^ proline, ITS + Premix universal Culture Supplement (Corning) (including 6.25 μg ml^−1^ insulin, 6.25 μg ml^−1^ transferrin, 6.25 μg ml^−1^ selenious acid, 1.25 μg ml^−1^ bovine serum albumin (BSA), and 5.35 μg ml^−1^ linoleic acid), 20 × 10^–6^ m of Rho-kinase inhibitor Y27632 (Axon Medchem), 100 ng ml^−1^ growth/differentiation factor 5 (GDF5) (PeproTech), 100 ng ml^−1^ BMP-2 (INDUCTOS), 10 ng ml^−1^ TGF-β1 (PeproTech), 1 ng ml^−1^ BMP-6 (PeproTech), and 0.2 ng ml^−1^ basic FGF-2 (R&D systems) (Mendes et al., 2016). The microspheroids were cultured in 1.5 ml of CM, and half of the media volume was changed every 3–4 days for up to 3 weeks.

### Microtissue fusion assay

As a proof of concept, the fusion process was monitored for two different (hPDC) cell pools and four different spheroid maturation levels on days 1 (D1), 7 (D7), 14 (D14), and 21 (D21).

#### Formation of spheroid pairs

For each condition, microtissues were collected from two wells after (gently) pipetting up and down several times. The suspension was collected in a 15 ml falcon tube, centrifuged at 1300 rpm for 40 s, aspirated, and resuspended in 1.5 ml of CM. The spheroid suspension was added to a new well, containing an agarose insert and a 1% (w/v) agarose border to prevent spheroid loss (next or underneath the insert) and insert movement/floating. This procedure is illustrated in Figure 4A.

#### Image acquisition

An automated stage scanning microscope, as described by Deckers et al., 2018, was used to monitor the spheroid fusion process. Using a bright-field objective with ×4 magnification, approximately 60 microwells were captured in the field of view of a single frame. A 3-point focus map was constructed for each well using extended focal imaging of three layers with an optimal spacing of 22 µm. For each data set, the acquisition was initialized with a delay of approximately 1 h to allow for initial spheroid contact and temperature equilibration. Based on experimental data (Hall et al., 2021), images were acquired over a period of 29 h, with a time interval of 5 min. During acquisition, the conditions were maintained at 37°C and 5% CO_2_ in a humidified incubator.

#### Histochemistry and immuno-histochemistry of fused constructs

After approximately 48 h of fusion, the constructs were fixed with 4% paraformaldehyde (PFA) at RT for 1 h, washed three times with phosphate-buffered saline (PBS), and stored in 1% (w/v) agarose. The samples were embedded in paraffin and sectioned at 5 µm, and histological analysis was performed according to a previously reported method of Alcian Blue staining (pH 1, Sigma-Aldrich, United States) with nuclear fast red (Lab Vision, United States) (Fernando et al., 2017). Immunohistochemistry was performed to detect collagen type II (Col-2). Briefly, antigen retrieval was performed by incubation in 0.02 M HCl containing 1 mg/ml pepsin, and quenching of endogenous peroxidase activity was performed with 3% H_2_O_2_ for 10 min. Next, sections were blocked in serum for 30 min and incubated overnight at 4°C with the primary antibody rabbit anti-collagen type II (Merck Millipore, AB761; dilution 1:25). Next, the slides were blocked and incubated with the secondary antibody horseradish peroxidase (HRP)-conjugated goat anti-rabbit (Jackson ImmunoResearch, United Kingdom; 111–035–003; dilution 1:500) for 30 min, and peroxidase activity was determined using 3,3′-diaminobenzidine (DAB) (K3468, Dako, United States), followed by counterstaining with hematoxylin (Sigma-Aldrich, United States). Stained histological sections were visualized using an Olympus IX93 inverted microscope equipped with a DP73 camera (Olympus, Belgium) and a ×40 bright-field objective.

### Fusion analysis pipeline

A software tool was developed and implemented in MATLAB^©^ 2019a (MathWorks, MA, United States) to process the acquired data sets. In particular, the Image Processing Toolbox^TM^ and Machine Learning Toolbox^TM^ were required. It is composed of three parts: (I) semi-automated selection of doublet candidates for fusion analysis and (II) automated segmentation and subsequent tracking of the selected doublets and (III) feature extraction.

#### Selection of doublet candidates

Doublet candidates can be selected manually or semi-automatically based on the first frame. Initially, all microwells of the first frame were detected as described by Deckers et al., 2018. In the case of manual selection, the user can highlight the microwells of interest, that is, the software selects the closest detected microwell. For the semi-automated selection, the following steps were performed:

(I) Based on visual inspection, all the detected microwells were manually labeled with 0 (no candidate), 1 (possible candidate), or 2 (candidate). Considering the 2D nature of the approach, spheroid pairs with some degree of overlap and/or large size differences were considered “possible candidates,” while spheroid pairs with too much overlap or a lot of single cells and/or debris were labeled as “no candidates.” Examples are shown in Supplementary Figure S1. (II) The microwells were segmented to extract the microtissues present (see the segmentation procedure for more information). (III) For each sample, a feature set was extracted (Supplementary Table S1). (IV) All labeled sets were grouped per day (D1, 7, 14, and 21), split into 80% training and 20% validation sets, and merged to obtain the final training and validation set. A random forest classifier (MATLAB^©^ 2019a; MathWorks, MA, United States) was trained to automatically classify the positive (1 and 2) and negative (0) samples. The following parameters were used for the classifier: number of trees: 60; number of predictors: 30; leaf size: 3; and cost [0 1; 5 0]. The accuracy, sensitivity, precision, and F1-score were computed (for the separate maturation levels as well) and represented as mean ± SD using 5-fold cross-validation.

#### Doublet segmentation

**FIGURE 2:**
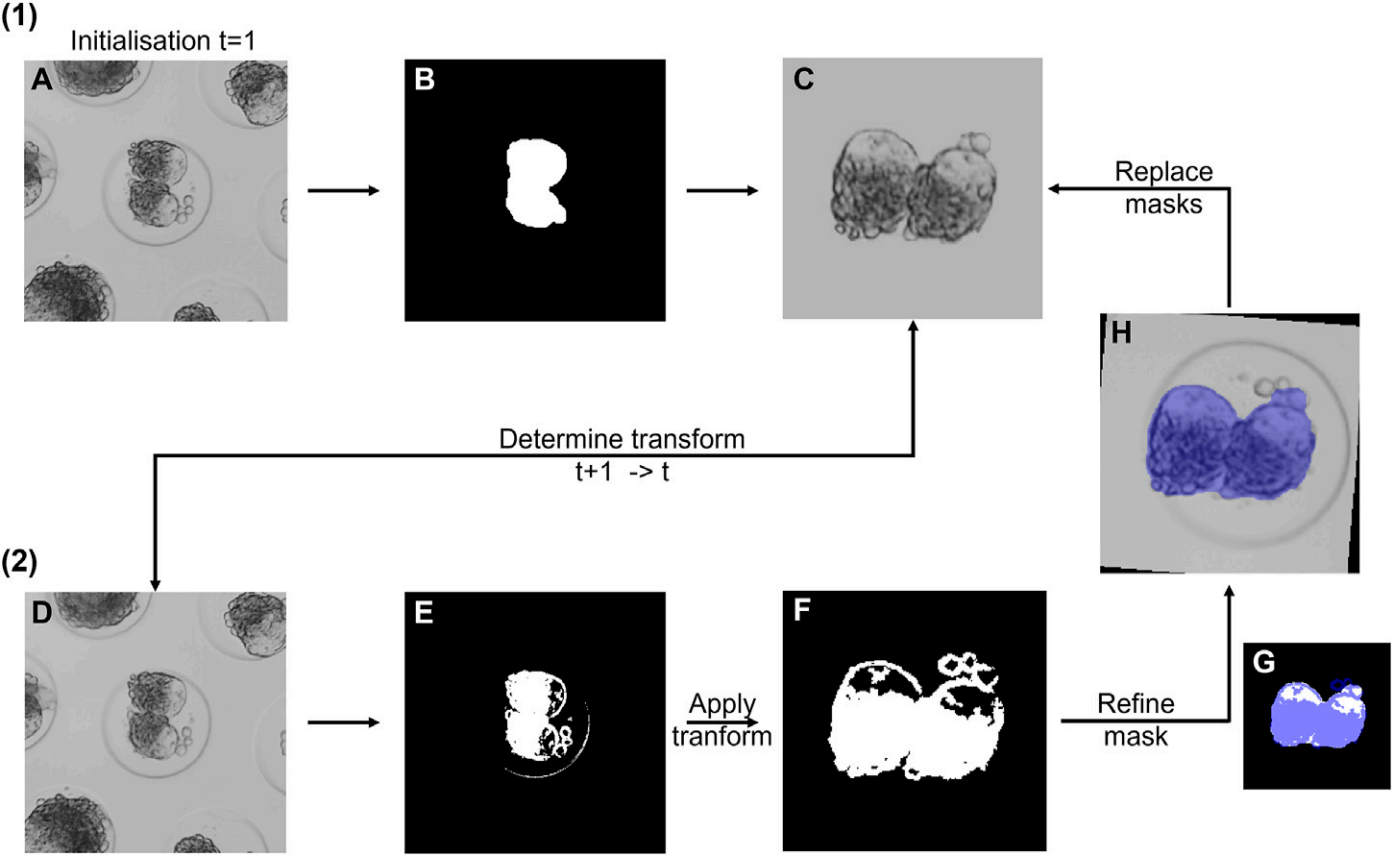
Overview of the doublet segmentation approach. (1) Initialization for frame 1. **(A)** Raw image. **(B)** Segmented mask. **(C)** Binary (for fine-tuning) and grayscale (for registration) mask. Only the grayscale mask is shown. (2) Segmentation of consecutive frames t + 1. **(D)** Raw image at time t + 1. The rigid transformation was determined with respect to the stored reference mask **(C)**. **(E)** Fine doublet segmentation. **(F)** Transformed mask. **(G)** Binary reference mask overlaid with the mask in **(F)**. **(H)** Refined mask overlaid on the raw image. Next, both reference masks of the previous time point were replaced with the new masks, and the cycle was repeated.

For all the selected doublets, the segmentation procedure in Figure 2 was initialized. For consecutive mask matching, doublets were tracked automatically over time. The raw image at time t + 1 was cropped around the previously detected microwell center (image t), circles were automatically detected, and the best candidate circle was selected. Consequently, the microwell coordinates were updated and stored. If no appropriate microwell was detected or it moved partly out of the field of view, the doublet sample was removed for further processing.

#### Feature extraction

**FIGURE 3:**
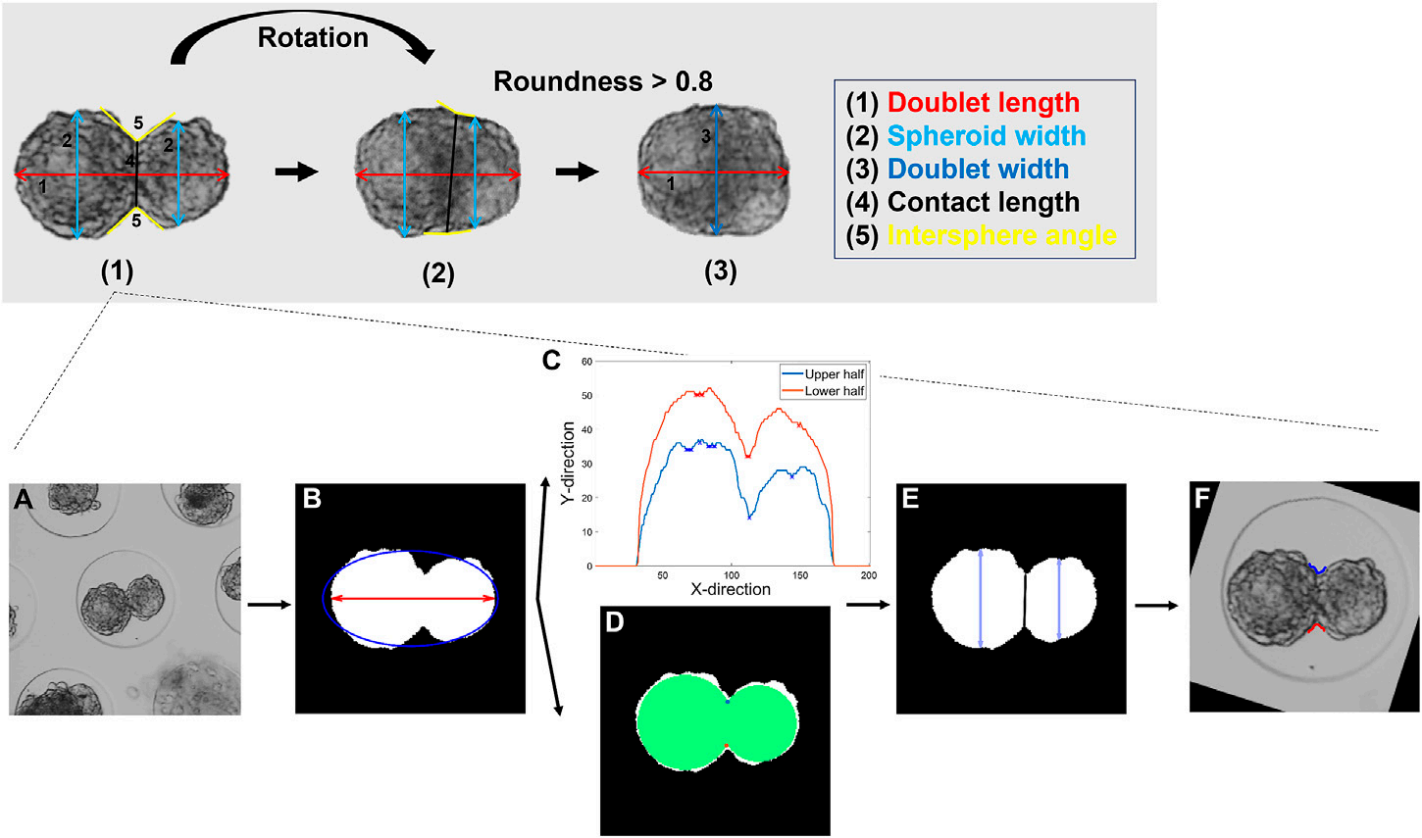
Description of the feature extraction procedure. Top panel: Visualization of the three different stages of the feature extraction with (1) the initialization for frame 1 and the subsequent feature extraction (2) before and (3) after a doublet roundness of 0.8 was reached. Bottom panel: Detailed representation of stage (1). **(A)** Raw image. **(B)** Segmented mask. The doublet area, length, and roundness were extracted. **(C)** Profile of the upper and lower half of the doublet with the detected minima. **(D)** Alternative method for the detection of the spheroid’s interface based on two maximally inscribed circles. **(E)** A cost function was defined to select the appropriate profile points. The spheroids were separated, and the contact length and both spheroid widths computed. **(F)** Upper and lower intersphere angles were extracted. (2) For subsequent frames, the same features as in (1) were extracted, and the rotation of the doublet with respect to the previous frame was determined. (3) As previously described, the mask was aligned with the horizontal axis. The doublet area, length, and width were extracted. More details on the features and the feature extraction are listed in the supplementary information (details on Panel 3 or Supplementary Table S2).

Several morphological features were extracted from the final masks. In Figure 3, the most relevant features are illustrated in a graphical overview. A feature matrix was stored for each individual doublet (Supplementary Table S2 for a complete list and definition).

### Quantitative validation of segmentation and feature extraction

The automated segmentation approach and the resulting feature extraction were compared to the measurements obtained by a human operator. The sample was manually segmented using the open-source function “imfreehand” (MATLAB^©^). Next, the doublet was horizontally aligned, and the doublet length, contact length, spheroid widths, and (local) intersphere angles were annotated by drawing straight lines. For both angles, two tangential lines, starting from the anchor point at the end of the contact length, were drawn to each side of the spheroid pair (Supplementary Figure S2).

The validation was performed on four different data sets (Y pool, D1-D7-D14-D21). For each set, six doublets were randomly selected from the candidates (see the paragraph on doublet selection) and manually labeled at fixed intervals of 25 min (50 min interval for D21) until appropriate fusion was achieved. Subsequently, the following measures were computed.

#### Sensitivity and precision of segmentation

The stored masks were used to compute the sensitivity (true positive rate, TPR) and precision (positive predictive value, PPV) according to Eqs 1, 2.

$$TPR=\;\frac{TP}{TP+FN}$$ (1)

$$PPV=\;\frac{TP}{TP+FP}$$ (2)

True positive (TP) is the number of correctly assigned pixels, false positive (FP) is the number of incorrectly identified pixels, and false negative (FN) is the number of missed pixels with respect to the manual segmentation.

#### Relative and absolute error of extracted features

In contrast to the automated approach, the doublet was not consistently aligned over time during the manual validation. Therefore, the spheroid widths and intersphere angles were averaged for comparison purposes. For all features, with the exception of roundness and averaged intersphere angle, the relative error was calculated according to Eq. 3:

$$Relative\;error\;\left(\%\right)=\;\left|\frac{MS-AS}{MS}\right|*\;100$$ (3)

where MS and AS are the values (units in S1 Table) obtained for each feature by performing manual (MS) and automatic (AS) segmentation, respectively. The absolute error was calculated for roundness and average intersphere angle. However, the rotation angle was not validated. The results were presented as mean ± SD.

### Feature processing on population and spheroid level

For each condition, 20–35 doublet samples were analyzed. In order to avoid the transfer of false positive samples to the analysis and obtain reliable results, doublet samples were randomly selected from the good candidate sets and analyzed according to the fusion analysis pipeline. Alternatively, doublet candidates can be automatically selected by the classifier and reviewed manually. All corresponding fusion videos were manually inspected for considerable doublet shifting or floating (out of the well), additional spheroids (through entering or not visible based on the first frame), and excess debris or single cells. Doublet samples that exhibited one of these events over time were removed.

The doublet area was normalized to the initial area, whereas the contact length was normalized with respect to the average initial (first 10 timepoints) spheroid width. The normalized contact length and averaged intersphere angle were plotted over time [mean ± standard error of the mean (SEM)] up to the point where 30% of the doublet samples had dropped out. Additionally, three video objects (five frames per second, Supplementary Material) were constructed to visualize the fusion response of individual doublets for certain conditions.

### Data-based modeling

For each sample, an exponential model was fitted (least-squares parameter estimation) to the doublet roundness response (Schötz et al., 2013; Brangwynne, Mitchison, and Hyman 2011):

$$Doublet\;roundness=plateau+b*e^{-\frac{t}{\tau }}$$

Physical limits were imposed on the plateau and b: [0.5 1] and [−0.6–0.1], respectively. The plateau represents the steady-state roundness, while the time constant (*τ*) represents the time (h) required for the roundness response to reach ≈63.2% of the plateau. The time constant and plateau are a measure for the fusion rate and quality, respectively.

Next, a linear model was fitted (least-squares parameter estimation) to the normalized contact length:

Normalized contact length=intercept+slope∗t

To ensure linear behavior, only data up to 5 h were considered (Figure 7). Physical limits were imposed on the intercept and slope: [0.05 1.4] and [0 1], respectively. The plateau, time constant (*τ*), and slope were extracted for further analyses.

### Statistical Analysis

All analyses were performed with at least 20 replicates per condition. Data were represented as mean ± SEM or box plots with boundaries determined using the inter-quartile range. In case the data were normally distributed and had equal variance (Bartlett’s test), they were compared with one-way ANOVA and Tukey-Kramer post hoc test. Otherwise, the Kruskal–Wallis (Breslow 1970) and Dunn–Sidak post hoc tests were used. The results were considered statistically different for *p-*values lower than 0.05 (*: *p* < 0.05; **: *p* < 0.01; ***: *p* < 0.001). Statistical analysis was performed using MATLAB (Mathworks, Inc., United States).

## Results

### The agarose microwell format for culturing microtissues and monitoring their fusion behavior

**FIGURE 4:**
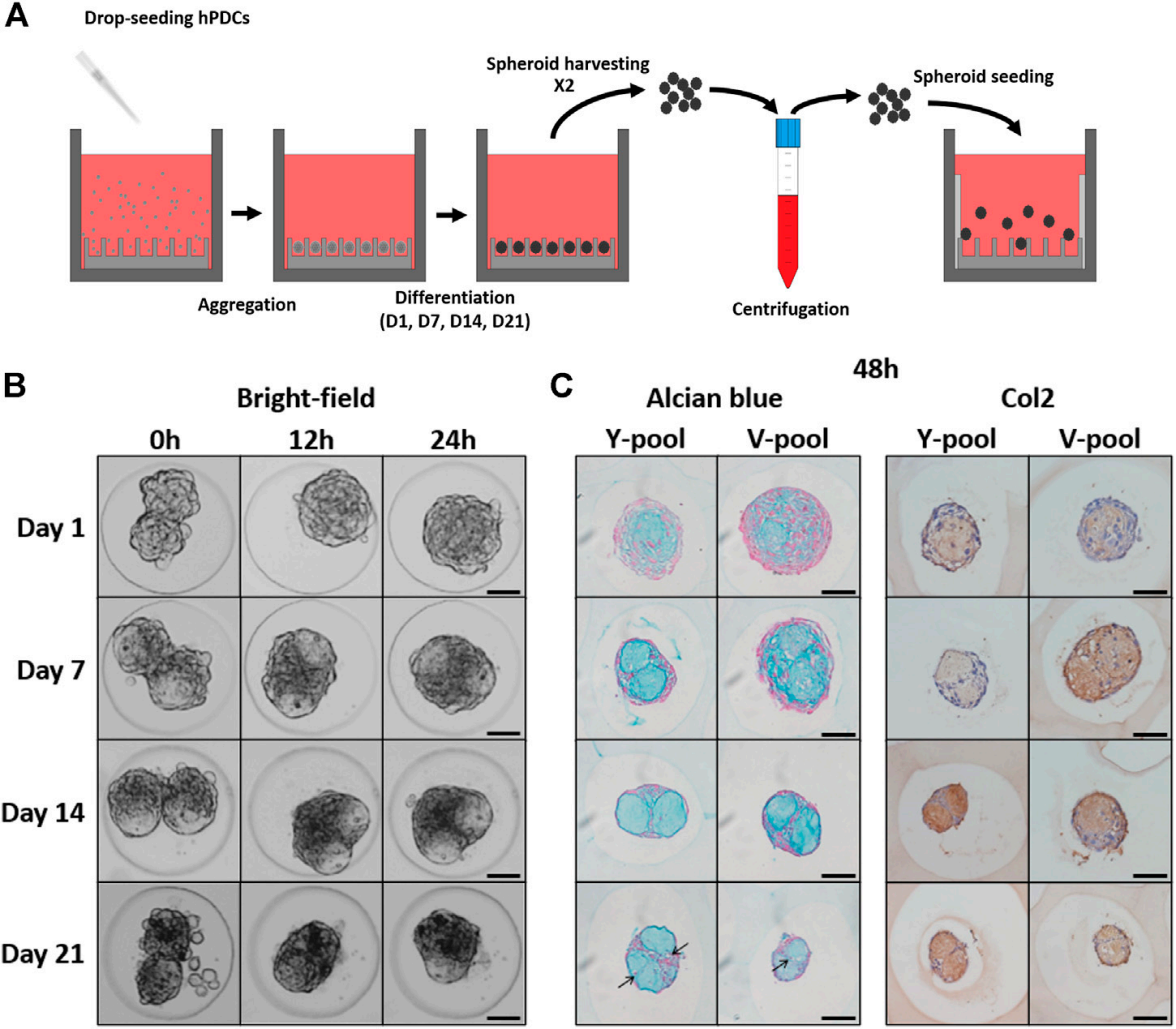
Spheroid fusion assay. **(A)** Schematic overview of the experimental procedure to obtain spheroid pairs. The main steps are cell seeding, cellular aggregation, differentiation, harvesting/centrifugation, and spheroid seeding. **(B)** Time-lapse images of the morphological response during spheroid fusion for all different maturation stages of the Y pool. **(C)** After 48 h, the fused constructs were stained for Alcian Blue (AB) and Collagen Type II (Col2). Scale bars: 50 µm.

The selected microwell platform enabled the production of a large number of homogeneously sized microtissues (Hall et al., 2020; Hall et al., 2021) and their subsequent fusion (Supplementary Figure S3). Spheroids composed of approximately 100 cells were seeded and cultured for up to 3 weeks in a chondrogenic differentiation medium, as previously reported by Leijten et al., 2016. Doublets were generated according to the procedure described in Figure 4A. During the maturation process, cell death occurs, resulting in an accumulation of debris in the microwells over time (Supplementary Figure S3A). Using our method, most cell debris was effectively removed. However, quite some single cells were observed for day 21 doublets (Figure 4B, Supplementary Figures S3A,B). All conditions exhibited active tissue fusion over time (Figure 4B, Y pool), indicating that viable microtissues with a high fusion capacity were produced. Transparent-like regions were also observed starting from day 7, which could indicate deposition of the extracellular matrix (ECM). Alcian blue staining, specific for glycosaminoglycan-rich ECM, and collagen 2 staining confirmed the deposition of cartilaginous ECM over time, with pre-hypertrophic-like chondrocytes visible after 23 (21 + 2) days of culture (Figure 4C, black arrows). Moreover, two separate matrix modules were visible starting on day 7, with more cells concentrated at the interface of the modules.

Furthermore, an important parameter of the culture format is the potential number of doublets that can be generated, that is, the doublet seeding efficiency. The number of selected doublets and the total number of detected microwells are listed for each condition in Supplementary Table S3. There is a large imbalance between the number of doublet candidates (candidates + possible candidates) and ‘no candidates’, affected by the maturation level and cell pool. In general, with the exception of D7, a decrease in the number of total doublet candidates was observed with an increased maturation level and a lower number of doublets for the V pool (with respect to the Y pool). On average 8.3% of the detected microwells contained a doublet for further analysis.

### Semi-automated selection of spheroid pairs with manual quality control for high-throughput screening applications

A general model including both cell pools and all maturation levels was established for the classification of doublet candidates. To retain a sufficient number of doublets for analysis, a higher cost was assigned to false negative classifications, whereas false positive samples could be discarded by the user. In Table 1, the performance of the classifier in terms of its accuracy, sensitivity, precision, and F1-score is reported. As previously mentioned, approximately 8.3% of the microwells contained doublets compatible with our approach, whereas an overall classification accuracy of 96.3% was obtained. There were also significant differences between the maturation levels, with averaged accuracies around 95.0–95.4% for D1 and D21, while average accuracies around 97.2% and 97.4% were obtained for D14 and D7, respectively. A lower percentage of doublets was retained by the classifier (sensitivity) as a function of maturation time, with a strong decrease for D21 samples. On average, approximately 50% of the doublet samples in the D21 test set were incorrectly discarded by the classifier. No clear trend was observed for the precision. In general, approximately one out of five doublets were wrongly classified as negative, while one out of four selected doublets were false positives and therefore should be manually removed. In Supplementary Table S4, examples of false positive and false negative samples, randomly selected from the classified test samples, are shown.

**TABLE 1:** Performance of the trained classifier. The classifier was evaluated in terms of its accuracy, sensitivity, precision, and F1-score on the whole test data set as well as on the separated test data sets for each individual maturation level. The classifier was validated using 5-fold cross validation (mean ± SD).

Maturation level	Accuracy	Sensitivity	Precision	F1-score
D1 (Y + V)	0.950 ± 0.007	0.920 ± 0.035	0.759 ± 0.026	0.832 ± 0.024
D7 (Y + V)	0.974 ± 0.009	0.882 ± 0.062	0.667 ± 0.091	0.756 ± 0.066
D14 (Y + V)	0.972 ± 0.007	0.842 ± 0.067	0.839 ± 0.034	0.840 ± 0.044
D21 (Y + V)	0.954 ± 0.006	0.515 ± 0.064	0.743 ± 0.056	0.608 ± 0.061
D1-D21 (Y + V)	0.963 ± 0.002	0.808 ± 0.026	0.760 ± 0.020	0.783 ± 0.013

### Quantitative assessment of the segmentation approach and feature extraction

The maturation process of spheroids presents some challenges such as transparent, ECM-like (low-contrast) regions, single cells, and cell debris. Therefore, the segmentation approach was validated for all maturation levels of the Y pool. In Table 2, the sensitivities and precisions of our approach are listed for each set. On average, values between 96.8% and 98.8% were obtained, with relatively small standard deviations. In general, our algorithm performed consistently across the different maturation levels.

**TABLE 2:** Sensitivity (TPR) and precision (PPV) of the automatic segmentation approach. Sensitivity and precision of the proposed methodology with respect to manual segmentation. For each validation set, the results are presented as mean ± SD, with N the number of samples per data set.

	D1	D7	D14	D21	Total
Roundness <0.8	*n* = 62	*n* = 126	*n* = 196	*n* = 98	*n* = 482
** **Sensitivity	0.9683 ± 0.0178	0.9768 ± 0.0146	0.9786 ± 0.0153	0.9791 ± 0.0096	0.9769
					± 0.0149
** **Precision	0.9880 ± 0.0067	0.9824 ± 0.0152	0.9769 ± 0.0099	0.9771 ± 0.0141	0.9798
					± 0.0127
Roundness >0.8	*n* = 138	*n* = 83	*n* = 75	*n* = 74	*n* = 370
** **Sensitivity	0.9727 ± 0.0139	0.9811 ± 0.0129	0.9792 ± 0.0195	0.9789 ± 0.0106	0.9771 ± 0.0148
** **Precision	0.9853 ± 0.0088	0.9808 ± 0.0092	0.9755 ± 0.0159	0.9802 ± 0.0084	0.9813 ± 0.0112

After segmentation, several features such as the doublet area, doublet roundness, and contact length were automatically extracted. It was assumed that spheroids could only be accurately separated up to a certain point. Therefore, the contact length and the corresponding intersphere angles were not monitored after a roundness of 0.8 was reached. The relative or absolute error values (mean ± SD) estimated for the different features are shown in Table 3. For the doublet area, length, and averaged spheroid width/doublet width, the algorithm obtained average relative error values within a small range of 1.16–2.27% for all data sets. For the contact length, the error values were larger (2.30–4.66%) and increased as a function of culture time. In general, the doublet roundness was characterized by relatively consistent absolute error values (approximately 0.01), whereas the subjective nature of the intersphere angle resulted in higher absolute errors. In general, no significant differences in the error rate were observed before and after a roundness of 0.8 was reached.

**TABLE 3:** Relative and absolute errors obtained for the different features with respect to manual feature annotation. The quantification was separated in function of the maturation time and the feature extraction procedure (before and after a roundness of 0.8), with the number of samples (N) indicated for each set. The results are represented as mean ± SD.

	D1	D7	D14	D21	Total
Roundness <0.8	*n* = 62	*n* = 126	*n* = 196	*n* = 98	*n* = 482
** **Doublet area (relative)	2.25 ± 1.97	1.54 ± 1.92	1.68 ± 1.22	1.47 ± 1.54	1.67 ± 1.61
** **Doublet length (relative)	1.26 ± 1.16	1.19 ± 1.17	1.18 ± 1.24	1.62 ± 1.87	1.28 ± 1.37
** **Doublet roundness (absolute)	0.0083 ± 0.0072	0.0107 ± 0.0102	0.0100 ± 0.0104	0.0102 ± 0.0100	0.0100 ± 0.0099
** **Contact length (relative)	2.30 ± 1.89	2.85 ± 2.30	2.75 ± 2.77	4.66 ± 5.01	3.11 ± 3.27
** **Averaged spheroid width (relative)	1.91 ± 1.93	1.57 ± 1.55	2.12 ± 1.81	1.80 ± 1.57	1.89 ± 1.72
** **Averaged intersphere angle (absolute)	12.27 ± 11.93	10.70 ± 8.47	10.19 ± 9.51	13.44 ± 10.94	11.25 ± 9.96
Roundness >0.8	*n* = 138	*n* = 83	*n* = 75	*n* = 74	*n* = 370
** **Doublet area (relative)	1.89 ± 1.48	1.60 ± 1.16	2.26 ± 1.97	1.23 ± 0.98	1.77 ± 1.48
** **Doublet length (relative)	1.56 ± 1.45	1.95 ± 1.73	1.43 ± 1.59	1.16 ± 1.02	1.54 ± 1.49
** **Doublet width (relative)	1.54 ± 1.38	1.54 ± 1.33	2.01 ± 1.92	2.27 ± 2.04	1.78 ± 1.66
** **Doublet roundness (absolute)	0.0111 ± 0.0112	0.0107 ± 0.0107	0.0135 ± 0.0140	0.0124 ± 0.0105	0.0118 ± 0.0116

Unlike in the manual segmentation process, the doublets were consistently aligned over time using the algorithm. This enabled the tracking of both spheroid widths (i.e., left and right spheroids) and intersphere angles (i.e., up and down), separately. In addition, the contact length was accurately monitored, even beyond the ability of a user to still separate the spheroids, by relying on the contact information from previous time points.

### Influence of maturation level on morphological changes and fusion kinetics of spheroid pairs

**FIGURE 5:**
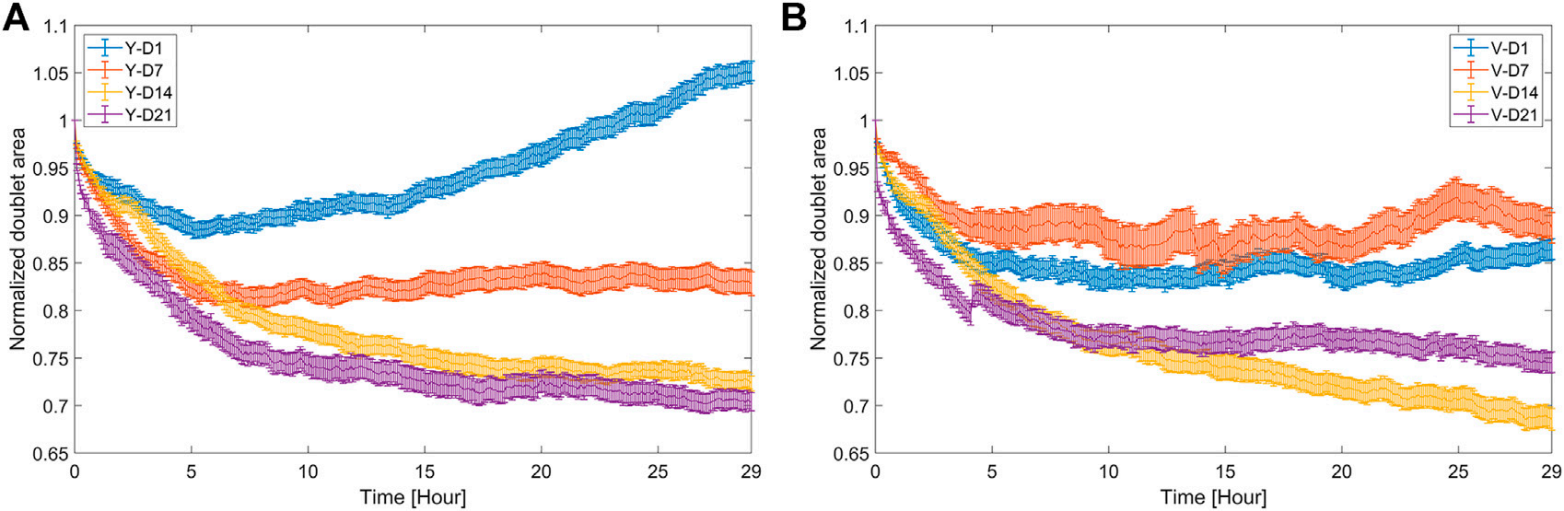
Area response of doublets during fusion. Doublet area normalized with respect to the initial area (mean value ±SEM, *n* = 20–35) for **(A)** Y and **(B)** V pool.

Doublet candidates were automatically segmented, and their features (as described in Supplementary Table S2) were extracted. As an example, the monitoring of a single doublet over time is shown in Supplementary Video S1. For each condition, 20–35 doublets were selected at random from the manually labeled candidate sets for thorough analysis. The most relevant features, that is, the doublet area and roundness, contact length, and intersphere angle, were visualized, and parameters related to the fusion rate and quality were extracted. As shown in Figure 5, the area response exhibited a distinct behavior in function of tissue maturity and the cell pool. Initially, the doublet area shrank under all conditions because of fusion. While the area of the D14 and D21 doublets decreased monotonically below 0.75, for the D1 and D7 doublets, the decrease in area was lower and followed by an increase or plateau. Particularly for Y-D1, there was a strong increase, where after 29 h, an average growth of 5% was observed with respect to the initial doublet area. Growth was expected for these relatively immature spheroids but could also still be present to a smaller extent at D14 and D21.

**FIGURE 6:**
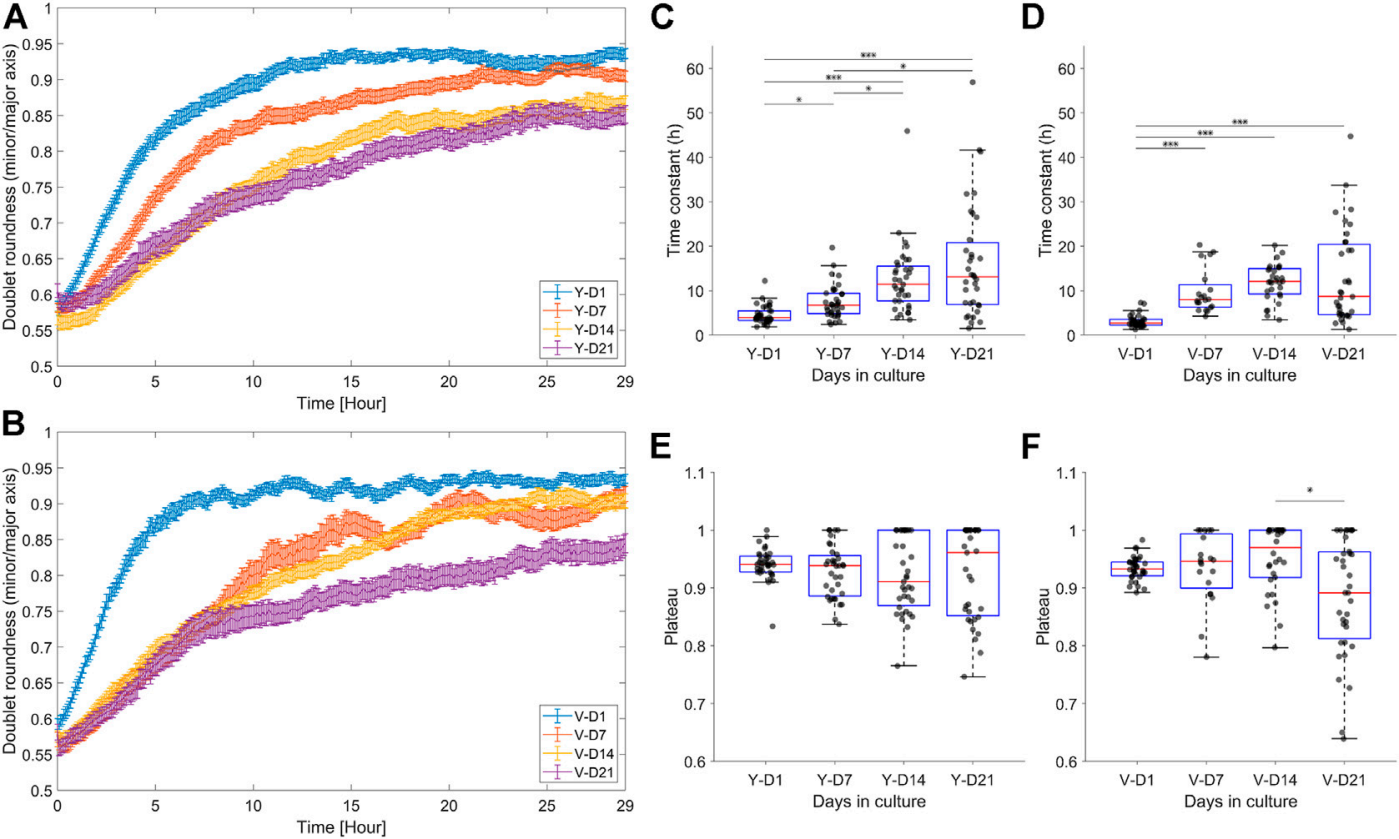
Dynamic response of the doublet roundness and the distribution of the extracted fusion parameters. **(A,B)** Average response ±SEM of the doublet roundness for the different levels of maturation, respectively, for the Y and V pools. **(C,D)** Box plot of the fusion rate parameter, i.e., the time constant, respectively, for the Y and V pools. **(E,F)** Box plot of the fusion quality parameter, i.e., the plateau, respectively, for the Y and V pools. Data were compared with Kruskal–Wallis and Dunn–Sidak post hoc tests. Significance was visualized with *p < 0.05; **p < 0.01; ***p < 0.001.

Doublet roundness is an important feature because it is dimensionless and therefore not directly influenced by an increase in doublet area. As shown in Figures 6A,B, the maturation level had a significant influence on the fusion process of both pools. For ease of interpretation, parameters such as the time constant and plateau were extracted from the time series (Figures 6C,D), relating to the rate and quality of fusion, respectively. Increased spheroid maturation slowed down the fusion rate of the spheroid pairs, exhibiting a gradual decrease for the Y pool (Figure 6C) and an abrupt decrease for the V pool at D7 (Figure 6D). For both pools, there was no significant difference in fusion rates between D14 and D21. The distribution of the fusion parameters broadened as a function of the maturation level (SD time constant – Y-D1: 2.07; Y-D21: 12.75; V-D1: 1.37; V-D21: 10.50, SD plateau – Y-D1: 0.0277; Y-D21: 0.0796; V-D1: 0.0205; V-D21: 0.1016, Figures 6E,F). For example, the number of doublets that exhibited an oval shape at steady state (i.e., a lower plateau) increased with maturation time, which could also be linked to the broader distribution of the time constant (especially for D21).

**FIGURE 7:**
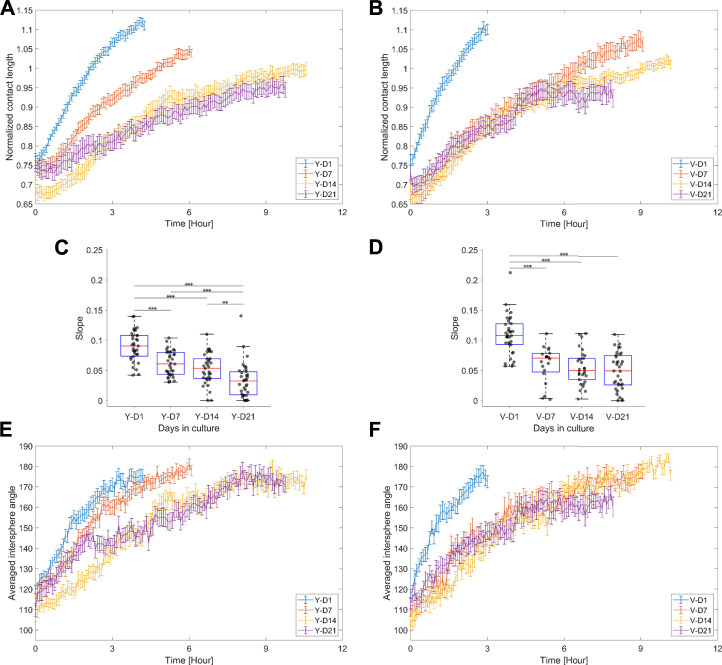
Normalized contact length and averaged intersphere angle. **(A,B)** Average response ±SEM of the normalized contact length for the different maturation stages, respectively, for the Y and V pools. **(C,D)** Box plots of the integration rate, i.e., the slope extracted from the linear model fit on the normalized contact length (data up to 5 h were considered), for the different maturation stages, respectively, for the Y and V pools. Data were compared with one-way ANOVA and the Tukey–Kramer post hoc test. Significance was visualized with *: *p* < 0.05; **: *p* < 0.01; ***: *p* < 0.001. **(E,F)** Average response ±SEM of the averaged intersphere angle for the different levels of maturation, respectively, for the Y and V pools. 20–35 doublet samples were analyzed per condition.

Figure 7 shows the dynamic responses of the normalized contact length and averaged intersphere angle. The normalized contact length can be interpreted as a connectivity measure between spheroids, and its increase is driven by the migration of cells across the spheroid modules. Initially, up to approximately 5 h, the rate at which the cells crossed the doublet interface was characterized by a linear response (Figures 7A,B). The slope parameter (Figures 7C,D) indicated a gradual or abrupt decrease in the integration rate with spheroid maturity for the Y and V pools, respectively, correlating with the doublet roundness trends (Figure 6). The intersphere angle can also be roughly interpreted as a measure of connectivity, where a value of 180° represents a smooth transition between spheroids (Figure 3F). Although the dynamic response of the intersphere angle was relatively noisy, a trend toward 180° was observed for each condition, but at a lower rate with increased maturation.

Other features, such as the doublet length, spheroid widths, and angular rotation, were also automatically extracted but were not discussed in this study. Doublet roundness was preferred over doublet length as a measure of fusion rate and quality since the response of the latter is related to both the growth and fusion behavior of the spheroids, which are difficult to decouple from each other. Additionally, the averaged angular rotation over time (Supplementary Figures S5A,B) and the average angular rotation per doublet (Supplementary Figures S5C,D) are shown for both pools. The doublets showed limited angular rotation over a 5 min time window, likely inhibited by the presence of the ROCK inhibitor in the chondrogenic medium (Susienka, Wilks, and Morgan 2016). However, overall, the D14 and D21 doublets exhibited a decrease in rotational activity with respect to D1 and D7 doublets. Last, in order to visualize some of the similarities and differences observed in the graphs, Supplementary Videos S2, S3 were constructed.

## Discussion

Characterizing and understanding the underlying processes of tissue fusion are essential for the development of successful bottom-up tissue engineering approaches. In this study, a method was developed to automatically monitor the fusion behavior of spheroid doublets. Because of the ease of fabrication, scalability, and formation of uniformly sized spheroids, agarose microformat was selected to produce and subsequently fuse the microtissues. As a proof of concept, immature spheroids and differentiated cartilage intermediate microtissues of different maturation, where the most mature microtissues are known for their bone-forming potential upon implantation *in vivo* (Hall et al., 2020), were produced and evaluated for their *in vitro* fusion potential. At all degrees of maturation, the doublets exhibited active tissue fusion and integration. Moreover, histological staining confirmed the gradual maturation of spheroids into microtissues, as previously reported (Hall et al., 2020).

An important aspect in spheroid fusion assays is the generation of a sufficient number of doublets. In one study (Susienka, Wilks, and Morgan 2016), a sandwich of two agarose inserts (Microtissues®) was formed, the assembly was centrifuged, and approximately 15–20 doublets were generated per gel (a seeding efficiency of 18–25%). Although a high efficiency was obtained, the accumulation of cell debris with culture time complicates the automated segmentation of the doublets. Moreover, additional handling operations can increase the risk of contamination. In most studies (Kim et al., 2018; Kosheleva et al., 2020), doublets were seeded by manually transferring individual spheroids, resulting in a theoretical doublet seeding efficiency of 100%. However, for large screenings, this approach is only feasible with an automated robotic platform (Hall et al., 2021). Our agarose platform enables high-throughput production of spheroids and has the potential to obtain a large number of doublet samples. Although lower doublet seeding efficiencies (ranging from 2.6% to 17%) were obtained compared to other seeding strategies (Susienka et al., 2016), a relatively high number of appropriate doublets were generated for each condition with minimal seeding effort. It is expected that this number can be further improved by reducing the number of overlapping spheroids through centrifugation of the well plate after spheroid seeding.

A doublet classifier can assist the user in the manual doublet selection. This would be useful for large-scale screening assays, especially if they contain many “negative” samples. In this study, a classifier with moderate performance was obtained. This is partly a result of the large variability among the samples of different data sets, imbalance between positive and negative samples, and uncertainty of manual labeling (see examples in Supplementary Figure S1). Overlapping and out-of-focus spheroids, single cells and debris, irregular-shaped spheroids, and so on are all factors that contribute to the complexity of the labeling process. This was also confirmed by looking at false positive and false negative classifications (Supplementary Figure S4). Moreover, the increased likelihood of certain events with maturation time can be correlated with a higher error rate for the D21 samples. Although the procedure is only semi-automated, it can still substantially reduce the time and effort required for the doublet selection. To the best of our knowledge, there is no software available yet to (semi-)automatically sample doublets under these variable circumstances. Therefore, we consider the constructed classifier already an important step toward a fully automated system.

Using automated, quantitative software tools for fusion assays, their throughput can be significantly increased, while yielding more consistent results. For our platform, the execution time of a doublet sample (all features except the rotation) was approximately 1.1s (normal desktop), whereas manual segmentation and feature annotation (Supplementary Figure S2) can take 80–100 s per sample. In the literature, doublet segmentation and/or subsequent feature extraction are often performed manually (Hajdu et al., 2010; Kim et al., 2018; Kosheleva et al., 2020; Omelyanenko et al., 2020) or only automated for a limited number of features (Susienka et al., 2016; Song et al., 2019). While the highest level of automation was reached by the system of Susienka et al., 2016 and an additional feature (i.e., fluorescence intensity) could be identified, it came at the cost of the use of invasive stainings. On the other hand, several systems for the automated analysis of single spheroids have been reported (Grexa et al., 2021; Piccinini 2015; Vinci et al., 2012). Although they achieve similar or higher performance than our method, they are often validated on large (>300 µm diameter) and/or relatively immature (<7 days) spheroids (Grexa et al., 2021; Piccinini 2015; Vinci et al., 2012). Small spheroids (<150 µm) and increased maturation levels (>7 days) pose additional challenges, such as single cells/debris and low-contrast regions. In our work, the abundance of cell debris was removed through a centrifugation step, while doublet registration over time dealt with additional noise (cells/debris) and low-contrast regions. On top of this, these methods do not quantify additional fusion features such as the contact length, both spheroid widths, the intersphere angles, and angular rotation. In many studies (Song et al., 2019; Kosztin et al., 2012; Flenner et al., 2008; Fleming et al., 2010; Messina et al., 2017), the ratio of the contact length to the spheroid radius/width is used to characterize the fusion behavior of spheroids. Therefore, the automated extraction of this feature is considered to be of great added value. For the averaged intersphere angle, the automated extraction was characterized by relatively high absolute errors. The intersphere angle is noise-sensitive and more prone to subjective interpretation, especially for small spheroid sizes. Therefore, we believe that this feature will be more reliable for larger spheroids.

As a proof of concept, the fusion process was examined for two independent cell pools at four different levels of maturation. For both cell pools, the trends observed in the area response of D1 and D7 doublets are plausibly a result of proliferation and/or ECM deposition, which counteracts the area decrease caused by fusion. Proliferation was previously demonstrated to be higher in D7 spheroids than in D14 and D21 spheroids (DNA and EdU from Hall et al., 2020). Moreover, the doublet roundness, contact length, and intersphere angle indicated a decrease in fusion capacity with increased *in vitro* pre-culture times. The accumulation of ECM with maturation time, visualized in the histological stainings, has previously been linked to a decrease in fusion capacity (Omelyanenko et al., 2020; Hall et al., 2021). On the other hand, an important driving force in tissue fusion is the migration of cells across spheroids at their interface, thereby reorganizing toward a round sphere. Hall et al., 2021 reported that the thickness of the outer cell layer and the spreading capacity of these cells decreased with maturation time. This could slow down or limit the reorganization toward a round structure, impacting the fusion rate and quality, respectively. In our data, a decrease in cell number at the periphery of the doublets was already observed at D7, and especially at D14 and D21. Moreover, a broadened distribution of fusion rate (time constant) and quality (plateau) with maturation time was observed for both pools. This is likely the result of an increase in biological and process variability, but additional experiments should be performed to verify this hypothesis. Similar trends in fusion behavior were observed for two independent cell pools, indicating that the relation between fusion rate (and capacity) and spheroid maturity could potentially be used to non-invasively estimate the maturation level of the cultured microtissues, as previously demonstrated by Hajdu et al., 2010.

Overall, the observed decrease in fusion rate and/or quality with maturation time is in accordance with the literature (Hall et al., 2021; Omelyanenko et al., 2020; Rago et al., 2009), illustrating the validity of our assay. Our method was validated on relatively small spheroids of hPDCs (<150 µm diameter) but could also be applied to larger spheroids after tuning the microwell diameter. In summary, a novel methodology is described, which will lead to a more streamlined and automated experimental setup for spheroid-based research and biofabrication (Moor et al., 2020; Park et al., 2021). The use of this methodology generates crucial data important for the study of spheroid/microtissue fusion and is expected to be applicable to other cell and tissue types, such as cancer (Vinci et al., 2012) and cardiac spheroids (Kim et al., 2018; Polonchuk et al., 2021). To the best of our knowledge, this is the first time that a platform for monitoring spheroid fusion has reached this level of automation.

## Conclusion

In conclusion, a versatile platform for monitoring spheroid fusion behavior was developed, compatible with large-scale analysis and allowing the automated extraction of a broad range of features for an in-depth analysis of spheroid fusion kinetics. Although the doublet selection procedure is still semi-automated, it significantly reduces the time spent on the selection of doublet candidates with respect to complete manual selection, especially for large assays and variable sample conditions. The potential of this approach was illustrated in a relevant biological case study, obtaining results that are in line with the literature. Further optimization of the experimental setup can improve the performance of the algorithm, and more automation can be achieved through the automatic removal of shifting doublets and other events. In this way, automated monitoring technologies can enable the use of high-throughput screening assays, which are necessary to unravel the mechanisms underlying microtissue fusion and eventually increase the success rate of the fabrication of functional tissue constructs. We envision that the approach could also be applied to other spheroid types and also to monitor, for example, the invasiveness of tumors in cancer biology.

## Data Availability

The raw data supporting the conclusions of this article will be made available by the authors, without undue reservation.
