# Supplementary material for: A platform for automated and label-free monitoring of morphological features and kinetics of spheroid fusion
Source: Front Bioeng Biotechnol. 2022 Aug 26;10:946992. doi: 10.3389/fbioe.2022.946992 (PMC9461702; doi:10.3389/fbioe.2022.946992)
Supplement: Supplementary file 3 [file DataSheet1.pdf]

Supplementary Material

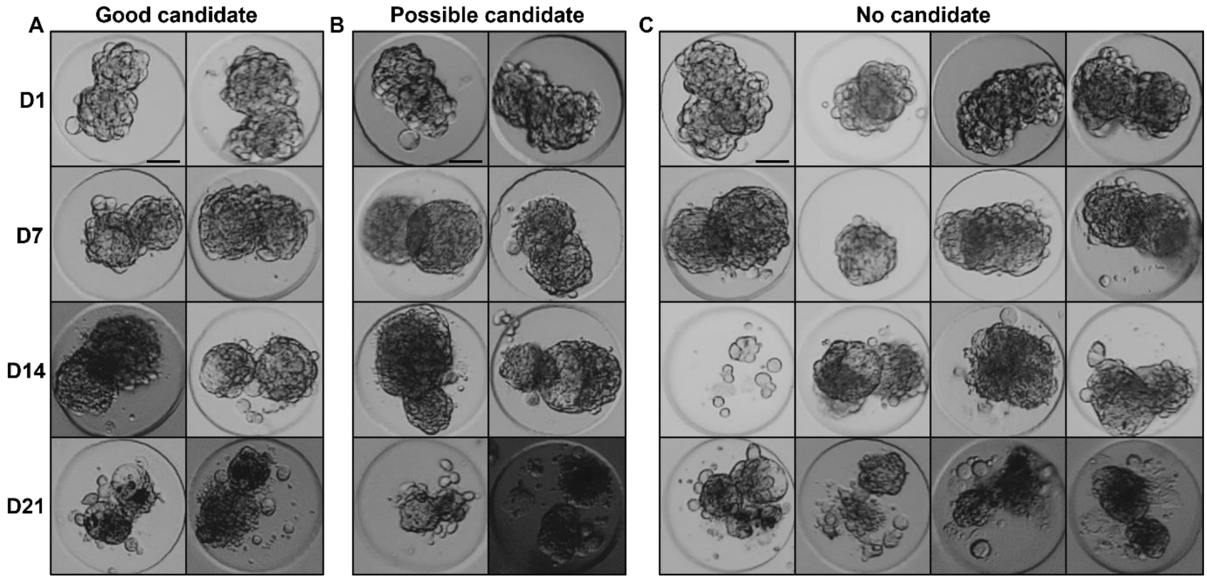

Supplementary Figure S1. Manual labelling of the detected microwells. Examples of **(A)** candidates, **(B)** possible candidates and **(C)** no candidates for the different maturation levels (D1, D7, D14 and D21) of both cell pools. Scale bars are 50 µm.

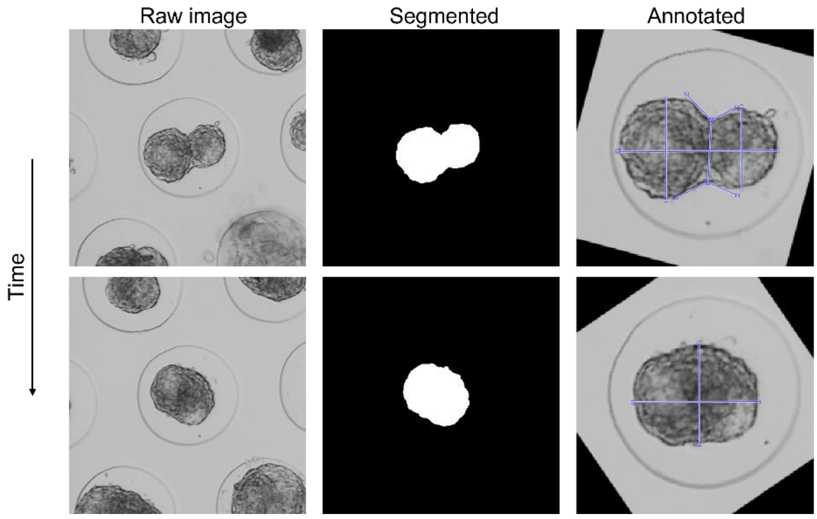

Supplementary Figure S2. Illustration of the manual segmentation and feature annotation over time.

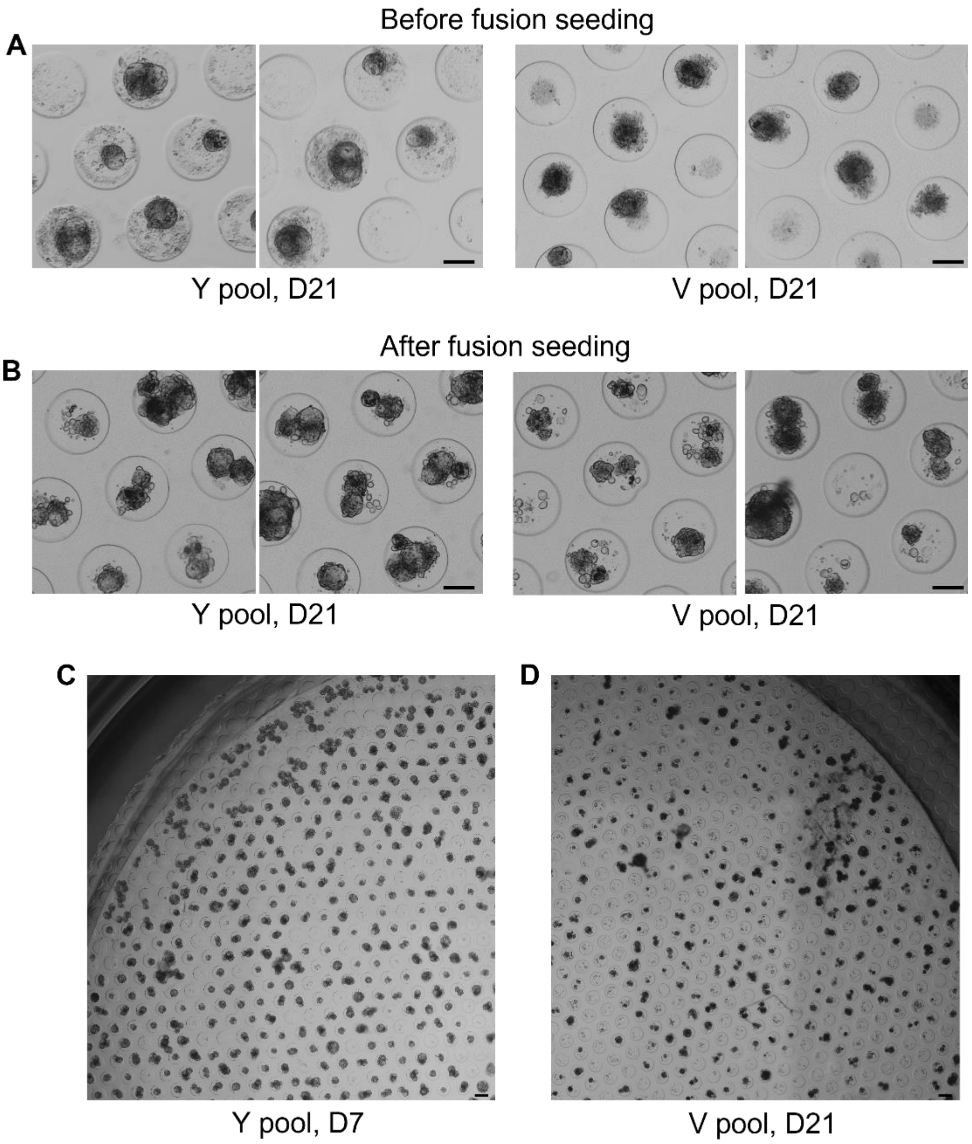

Supplementary Figure S3. Spheroids prior and after fusion seeding. (A) Y and V pool spheroids after 21 days of culture in chondrogenic differentiation medium. **(B)** Y and V pool spheroids seeded for spheroid fusion after 21 days of culture in chondrogenic differentiation medium. Scale bars are 100 µm. Overview image of seeded spheroids for the generation of doublets after **(C)** 7 days and **(D)** 3 weeks in chondrogenic differentiation medium. ¼th of the entire well overview image was shown. Scale bars are 200 µm.

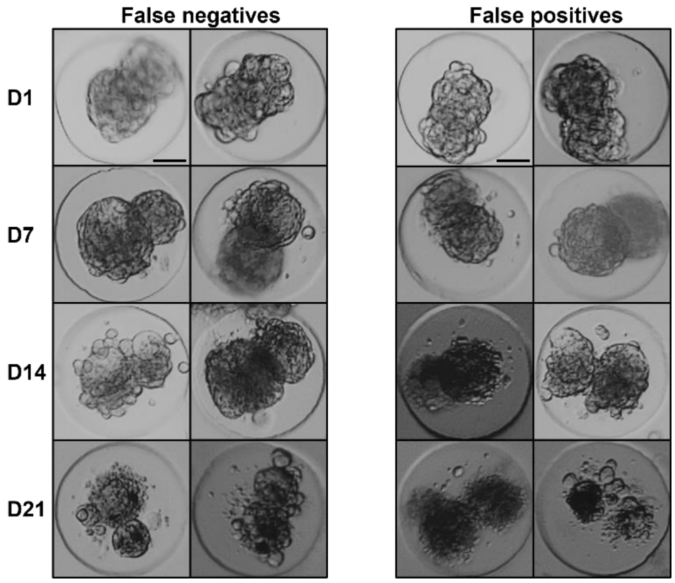

Supplementary Figure S4. Visualisation of wrongly classified samples by the doublet classifier. Examples of false negative (FN) and false positive (FP) samples for the different days (D1, D7, D14 and D21) and pools (Y and V pool, not indicated). Scale bars are 50 µm.

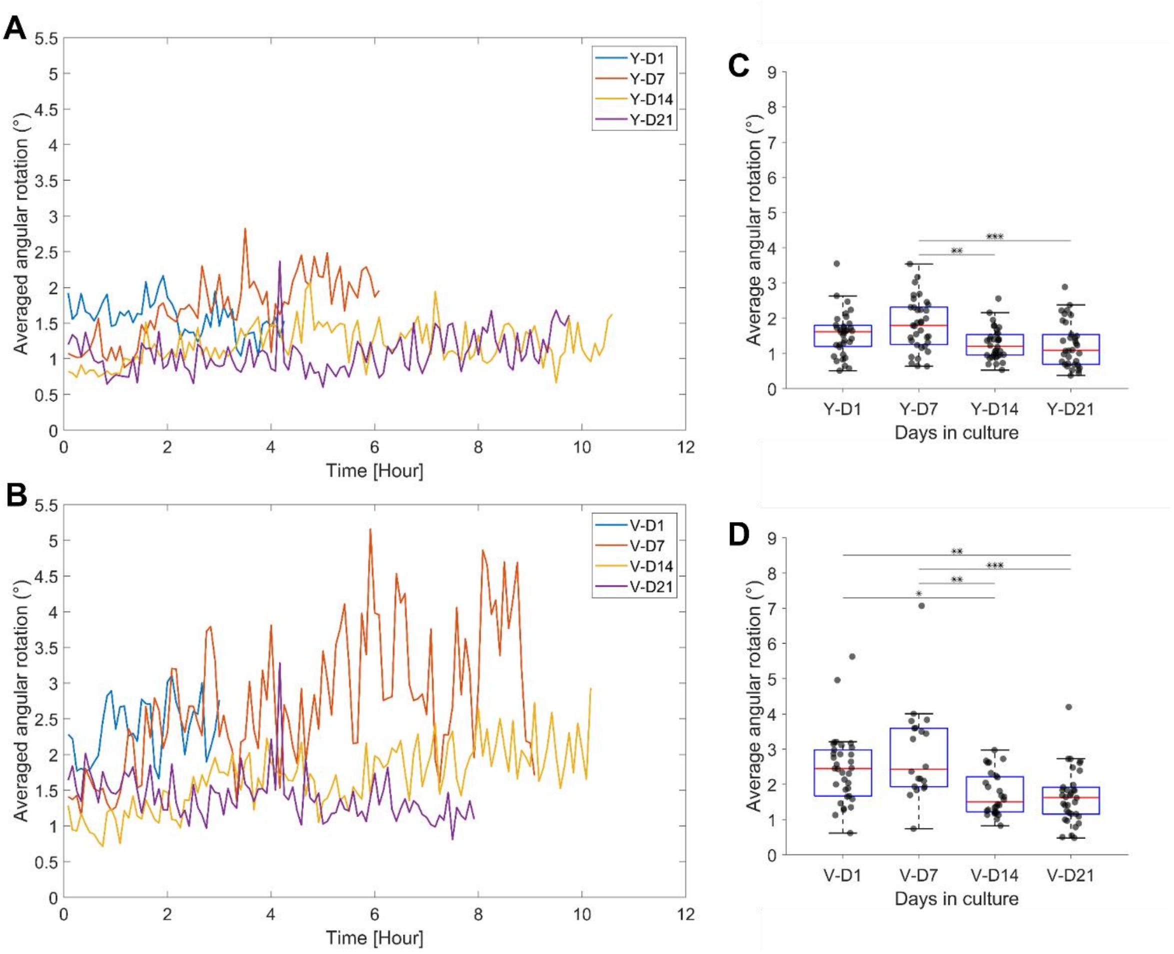

Supplementary Figure S5. Angular doublet rotation (n= 20-35 doublet samples). (A),**(B)** Averaged angular rotation over time, respectively for the Y and V pool. **(C)**,**(D)** Average angular rotation per doublet sample, respectively for the Y and V pool. Each data point represents an individual doublet. Data were compared with **(C)** one-way ANOVA and Tukey-Kramer post hoc test or **(D)** Kruskal-Wallis and Dunn-Sidak post hoc test. Significance was visualised with *: $p < 0.05$; **: $p < 0.01$; ***: $p < 0.001$.

Supplementary Table S1. Feature set extracted for each sample for the classification of doublets.

Feature index	Feature	Description
1:156	Generic Fourier Descriptors (GFD)[1]	Radial frequency (M) is 3 and angular frequency (N) is 12, resulting in 52 features. These features were extracted for the binary image of the doublet and both separated spheroid regions. Therefore, 156 (3x52) features were obtained per sample. The feature extraction was implemented using a Matlab function[2].
157:159	Roundness	Minor/major axis length of the ellipse that has the same normalized second central moments as the region. Computed for the whole and both separated regions.
160	Diff in roundness	Absolute difference in roundness between both separated regions.
161:163	Circularity	Circularity = (4*Area*pi)/($Perimeter^2$). Computed for the whole and both separated regions.
164	Diff in circularity	Absolute difference in circularity between both separated regions.
165:166	Area	Area [Pixels] of both separated regions.
167	Diff in area	Absolute difference in area [Pixels] between both separated regions.
168:174	Overlap	Based on the maximally inscribed circle method (described in Figure 3), multiple features relating to the overlap were calculated:<br>- Intersection over Union (IoU).<br>- Number of overlap pixels between both circles respectively divided by the number of pixels in circle 1, circle 2 and the merged circles.<br>- For the whole and both separated regions, the number of region pixels not overlapping with their corresponding inscribed circles, divided by the number of pixels in that region.
175	Contact ratio	The contact length (as described in Figure 3) over the averaged spheroid width.
176:178	Focus measure	Diagonal Laplacian [3]. The measure was implemented in Matlab by [4], but adapted to only consider the specified region. Computed for the whole and both separated regions.
179:187	Correlation measure	The gray-level co-occurrence matrix was computed with the following offsets: [0 1; 0 2; 0 3]. The graycoprops (Matlab) function was used to extract the 'correlation' measure. Computed for the whole and both separated regions.

Supplementary Table S2. Description of the features extracted from the doublets. For a more detailed explanation of the different features, have a look at the 'regionprops' documentation on the MathWorks website or Figure 3.

Main features	Description/extraction method [Unit]
File index	Frame number of the image sequence.
X Centroid	Horizontal coordinate (x-coordinate) of the doublet its center of mass (regionprops – 'Centroid').
Y Centroid	Vertical coordinate (y-coordinate) of the doublet its center of mass (regionprops – 'Centroid').
Doublet area	The number of pixels in the doublet region [Pixel] (regionprops – 'Area').
Doublet length	The average number of pixels around the center of mass of the doublet along the horizontal axis [Pixel].
Doublet width	The average number of pixels around the center of mass of the doublet along the vertical axis [Pixel]. The doublet width is only considered after a doublet roundness of 0.8 is reached.
Doublet roundness	The doublet roundness was computed according to the following formula: $$Roundness = \frac{Minor\ axis\ length}{Major\ axis\ length}$$ Minor/major axis length (regionprops – 'MinorAxisLength'/'MajorAxisLength') of the ellipse that has the same normalized second central moments as the doublet region. The value ranges from 1 (circle) to approximately 0 (infinite line segment).
Contact length	The length of the contact region, i.e. interface between the two spheroids [Pixel].

Upper and lower intersphere angle	The angles between the spheroid pair, using the edges of the contact length as anchor points [Degree]. Angles were extracted on both sides of the doublet and averaged to represent the averaged intersphere angle.
Rotation	Difference in doublet orientation between two consecutive frames [Degree].**
Width of left and right spheroid	The spheroid widths were computed around the center of mass of both spheroids, perpendicular to the doublet length [Pixel].* The widths were averaged to represent the averaged spheroid width.
Area of left and right spheroid	The number of pixels in the separated spheroid regions [Pixel] (regionprops – 'Area').*
Doublet orientation	The angle (θ) between the horizontal axis and the major axis of the ellipse that has the same normalized second central moments as the doublet region. The value ranges from -90° to 90° [Degree] (regionprops – 'Orientation').

* Since every doublet is composed of two spheroids, this feature is calculated for both spheroids.
** Can only be determined up to 180 degrees. A small time interval for imaging will limit the rotation of the doublet between consecutive frames.

Supplementary Table S3. Number of selected doublet candidates with respect to the total number of detected microwells for all conditions.

**Pool**	Y pool				V pool				
**Maturation level**	D1	D7	D14	D21	D1	D7	D14	D21	Total
# of candidates/ '2' doublet samples	210	65	171	118	111	25	71	69	840
# of possible candidates/ '1' doublet samples	86	55	73	54	58	24	20	19	389
# of detected microwells	1742	1916	1933	1851	1700	1905	1886	1899	14832
% of doublets	17,0%	6,3%	12,6%	9,3%	9,9%	2,6%	4,8%	4,6%	8.3%

Supplementary Video S1: Visualising the dynamic behaviour of the most relevant fusion features for a single doublet. In the top image, the raw image is shown overlaid with its segmentation mask. In the bottom image, the extracted features are indicated on the raw image. In the left and right panel, the dynamic response of the different features, as indicated on the y-axis, is shown.

Supplementary Video S2: Visualisation of the differences in spheroid fusion kinetics as a result of tissue maturation (D1 on top; D21 on bottom) for the V pool. In the center panel, the raw images (overlaid with their segmented masks) of both conditions are shown. In the left and right panel, the dynamic response of the different features are shown for both conditions, plotted with their respective mask colour.

Supplementary Video S3: Visualisation of the spheroid fusion response of both pools (Y pool on top; V pool on bottom) with the same level of tissue maturity (D7). In the center panel, the raw images of both conditions are shown. In the left and right panel, the dynamic response of the different features are shown for both conditions, plotted with their respective mask colour.

Supporting materials and methods

Detailed information on Figure 2

(A) The original image was cropped around the detected microwell, resulting in the raw image with the doublet candidate in the center of the image.
(B) The doublet was segmented from the raw image by combining (OR operation) Roberts gradient operator (threshold = 0.045) [5] and intensity thresholding (derived from Otsu's threshold)[6]. The gradient output was not normalised. The extracted region was overlaid (AND operation) with the binary micro-well mask (radius of 79 pixels), holes smaller than 600 pixels (4-connectivity) were filled and the mask was eroded with a disk of 10 pixels radius. In order to remove small regions, regions smaller than 300 pixels were filtered out and the mask was dilated with a disk of 10 pixels radius. Finally, the largest object was retained.
(C) The mask obtained in **(B)** was centred, horizontally aligned (based on the fitted ellipse that has the same normalized second central moments as the doublet region) and stored as a binary and grayscale reference mask. The background region was filled with constant grey values.
(D) A rough doublet segmentation was performed (according to **(B)**), the resulting mask was centred, aligned with the horizontal axis and stored as a grayscale mask. The grayscale mask was rotated an additional 180 degrees and for both masks, the rigid transformation with respect to the stored grayscale reference mask **(in C)** was determined. Based on a cross-correlation measure, the grayscale mask with the highest correlation to the previous (time-point) reference mask was retained and the corresponding transform was selected.
(E) A fine segmentation was performed (gradient threshold of 0.065 instead of 0.045 in **(B)**), without any postprocessing operations such as filling holes etc.
(F) The transform (determined in **(D)** was applied to the binary mask and the largest object was retained.
(G)-(H) The transformed mask was refined with respect to the previous (frame t) binary reference mask. Matching and small non-matching (up to 20 pixels) holes were filled, while larger non-matching holes were excluded. Moreover, 'missed' regions (with respect to the reference mask) larger than 25 pixels were extracted, 'smoothed' with the doublet border, and it was attempted to correct for under-segmentation based on the connectivity of these 'missed' regions with the doublet mask. Regions that were substantially (larger than 80%) connected to the doublet mask were retained and added to the doublet mask. Next, the previous operations **(B)** on erosion, removal of small regions and dilation were performed. Only the largest object (i.e. the doublet) was retained.
The final doublet mask was re-aligned with the horizontal axis (based on the fitted ellipse) and replaces the binary and grayscale reference mask of the previous time-point in **(C)**. The cycle was repeated for the next time point.

Detailed information on Figure 3

Stage (1):
(A) /

(B) The area of the doublet mask was extracted (regionprops – 'Area'). An ellipse was fitted with the same normalized second central moments as the doublet region, and its minor and major axis length (regionprops – 'MinorAxisLength'/'MajorAxisLength') were used to compute the doublet roundness. Next, the orientation (regionprops – 'Orientation') of the ellipse was used for alignment with the horizontal axis, and the doublet length was computed by averaging the lengths (along the horizontal axis) within a 2-pixel neighbourhood of the center of mass.

(C) The doublet was separated in an upper and lower half (along the horizontal axis) and all minima were detected for both profiles. Their location and prominence was stored.

(D) The aligned doublet region was vertically splitted in two separate (spheroid) regions and by maximising the minimum roundness of the separated regions, a rough separation was obtained. Next, a maximally inscribed circle was computed for each region, maximising the overlap with the respective region. The points where both circles intersected were also included as profile points in **(C)**. In case the circles initially did not intersect, they were both grown until intersection. The prominence of these points was set at 1.

(E) In order to obtain the final separation of the spheroids, the anchor (contact) point on the upper and lower half of the doublet was selected. All possible pairs of anchor points were considered and the pair with the lowest cost, according to a cost function based on the spheroids their circularity, prominence of the anchor points and shift between the upper and lower anchor point, was selected. The contact length was computed as the Euclidean distance between both anchor points. After separation, both spheroid areas (regionprops – 'Area') and widths were computed. The spheroid widths were computed by averaging the lengths (along the vertical axis) within a 2-pixel neighbourhood of the center of mass of the spheroid region.

(F) The upper and lower intersphere angle were measured through extraction of the local spheroid border (4 in total, 2 up and 2 down), starting from the anchor points of the contact length. The number of pixels considered for each border was 40% of the horizontal distance from the anchor point to the center of mass of the respective spheroid, with a minimum number of 10 pixels. Next, a linear function was fitted to each local border, with the anchor point as a fixed point. In this way, the linear functions were used to determine the upper and lower intersphere angle.

Stage (2): In order to determine the anchor points in the subsequent frames, the doublet was separated in an upper and lower half (along the horizontal axis, as in **(C)**) and all minima (with a minimum prominence of 2) were detected for both profiles. Their location and prominence was stored. Based on their prominence and horizontal distance to the averaged anchor point coordinate (based on previous frames), certain minima were retained. In addition to this, the averaged anchor points (upper and lower half) were also retained. All possible pairs (upper with lower half) were matched and the pair with the lowest Euclidian distance (minimal contact length) was selected as the new anchor points. The other features were extracted as described in **(1)**.

Stage (3): As previously described, the doublet length and width were extracted around the center of mass of the doublet, respectively along the horizontal and vertical axis of the horizontally aligned doublet region.

References

1. Zhang D, Lu G. A comparative Study of Fourier Descriptors for Shape Representation and Retrieval. Proc 5th Asian Conf Comput Vis. 2002; 646–651.
2. Kratzert F. FD = gfd(bw,m,n) - Implementation of the Generic Fourier Descriptors. In: MATLAB Central File Exchange [Internet]. 2021. Available: https://www.mathworks.com/matlabcentral/fileexchange/52643-fd-gfd-bw-m-n-implementation-of-the-generic-fourier-descriptors
3. Thelen A, Frey S, Hirsch S, Hering P. Improvements in shape-from-focus for holographic reconstructions with regard to focus operators, neighborhood-size, and height value interpolation. IEEE Trans Image Process. 2009;18: 151–157. doi:10.1109/TIP.2008.2007049
4. Pertuz S. Focus Measure. In: MATLAB Central File Exchange [Internet]. 2021. Available: https://www.mathworks.com/matlabcentral/fileexchange/27314-focus-measure
5. Roberts LG. Machine Perception Of Three-Dimensional Solids. Massachusetts Institute of Technology. 1963.
6. Otsu N. A Threshold Selection Method from Gray-Level Histograms. IEEE Trans Syst Man Cybern. 1979;9: 62–66.
